# Exposure to omentum adipose tissue conditioned medium from obese pregnant women promotes myometrial artery dysfunction

**DOI:** 10.1111/jog.13482

**Published:** 2017-10-13

**Authors:** Christina E. Hayward, Elizabeth J. Cowley, Colin P. Sibley, Jenny E. Myers, Mark Wareing

**Affiliations:** ^1^ Maternal and Fetal Health Research Centre, Developmental Biology and Medicine, Faculty of Biology, Medicine and Health University of Manchester Manchester UK; ^2^ St. Mary's Hospital Central Manchester University Hospitals NHS Foundation Trust, Manchester Academic Health Science Centre Manchester UK

**Keywords:** adipokines, adipose tissue, body mass index (BMI), obesity, pregnancy

## Abstract

**Aim:**

Underlying mechanisms of poor pregnancy outcome in obese (OB) mothers (body mass index [BMI] ≥ 30 kg/m^2^) are unknown. Our studies demonstrate that OB pregnant women have altered myometrial artery (MA) function related to the thromboxane and nitric oxide pathways. In obesity, increased central fat mass is associated with an altered endocrine milieu. We tested the hypothesis that in OB pregnant women the omentum, a central fat store, releases factors that promote dysfunction in normal MAs.

**Methods:**

Myometrial and omental adipose tissue biopsies were obtained from women with uncomplicated term pregnancies. Omental adipose tissue explants from six normal weight (NW; BMI 18.5–24.9 kg/m^2^) and six OB (BMI ≥ 30 kg/m^2^) women were cultured and the conditioned medium collected and pooled to produce NW medium and OB medium. Adipokine concentrations were measured using enzyme‐linked immunosorbent assays. Wire myography was used to assess the effect of conditioned medium (NW or OB; N = 7) or leptin (100 nM; N = 5) exposure on MA responses to U46619 (thromboxane‐mimetic) and bradykinin (endothelial‐dependent vasodilator).

**Results:**

OB medium had higher leptin and lower adiponectin levels than NW medium. U46619 and bradykinin concentration response curves shifted upwards in MAs exposed to OB medium but were unaffected by leptin.

**Conclusions:**

Omental adipose tissue from OB pregnant women produced altered concentrations of adipokines. Acute OB medium exposure induced MA dysfunction, an effect not mirrored by exposure to leptin. These data suggest that an aberrant endocrine environment created by increased central adiposity in OB pregnant women induces vascular endothelial dysregulation, which may predispose them to a poor pregnancy outcome.

## Introduction

Obesity (body mass index [BMI] ≥ 30 kg/m^2^) is a significant risk to global health as it increases the risk of developing chronic cardiovascular disease and metabolic disorders, such as diabetes. Rapidly escalating rates of obesity observed in the general population are mirrored in the pregnant population; globally over 100 million women of child bearing age are affected.^1^, ^2^ In the United Kingdom, the national rate of obese (OB) women registering for antenatal care has doubled to 16% over the last 20 years.^3^, ^4^, ^5^ Obesity has important ramifications for the health and wellbeing of an expectant mother, as well as that of her developing baby. Obesity is an independent risk factor for pregnancy complications, including pre‐eclampsia (PE, a pregnancy‐specific cardiovascular disorder characterized by maternal hypertension and proteinuria), and poor obstetric outcomes, such as aberrant fetal growth (fetal growth restriction and fetal overgrowth) and intervention in labor (increased likelihood of cesarean section);^6^, ^7^ however, why this occurs is unknown. Development of PE and aberrant fetal growth are also associated with chronic health problems (e.g. cardiovascular disease, obesity and diabetes) for both mother and baby (in adulthood).^8^

Our previous studies demonstrate that, in OB pregnant women, myometrial artery (MA) function is impaired compared to MAs in normal weight women.^9^ Furthermore, this was not a global dysregulation in MA function but appeared specific to the thromboxane and nitric oxide (NO) regulatory pathways. The mechanisms underpinning impaired contraction and vasodilation in OB pregnant women remain unclear.

The underlying mechanism of vascular dysfunction in OB mothers might arise from increased visceral adipose tissue mass. Adipose tissue normally produces and releases numerous adipokines, signaling factors that mediate a wide range of physiological processes, including vascular reactivity.^10^, ^11^ Adipokines are produced abundantly from visceral white adipose tissue including the omentum, a fat pad that covers the intestines.^12^ This central fat store expands in obesity as a result of hypertrophy and/or hyperplasia. This expansion is associated with increased systemic circulating concentrations of prothrombotic, proinflammatory and vasoactive factors (e.g. leptin, tumor necrosis factor [TNF]‐alpha, plasminogen activator inhibitor 1 and interleukins [IL]‐6 and IL‐8) and decreased levels of vasoprotective adipokines, such as adiponectin.^10^, ^13^, ^14^, ^15^

Aberrant circulating adipokines may promote the systemic, utero‐placental and feto‐placental vascular dysfunction evident in OB mothers. For example, hyperleptinemia, a state associated with OB individuals, promotes an NO bioavailability imbalance and increased oxidative stress in the systemic circulation.^16^ Additionally, the presence of leptin receptors on vascular endothelial cells demonstrates an ability of vascular tissues to respond to fluctuations in circulating leptin levels.^17^ Previous data suggest that adipokines (e.g. leptin, TNF‐alpha, IL‐6) produced from cultured human adipocytes can impair human umbilical venous endothelial cell function by upregulating monocyte adhesion, a mechanism associated with vascular disease.^18^

As obesity rates are escalating, it is important to understand what underpins impaired vascular function, which might contribute to the susceptibility of OB mothers to poor pregnancy outcome. The aim of this study was to determine whether the omentum, a central fat store, releases factors that modify endothelial function in maternal MAs. We tested the following hypotheses: (i) omental adipose tissue from OB pregnant women releases altered concentrations of adipokines and cytokines compared to normal weight (NW) pregnant women; and (ii) in an *in vitro* bioassay, altered levels of adipokines released from omental adipose tissue impair MA function.

## Methods

### Ethical information

The NRES North West Haydock Ethics Committee (Ref: 08/H1010/55) approved this study and written informed consent was obtained from all participants prior to delivery. This investigation conformed to the principles outlined in the Declaration of Helsinki.

### Participants and tissue collection

Omental adipose tissue and/or myometrial biopsy specimens, obtained from the upper part of the lower segment uterine incision, were collected from women with uncomplicated pregnancies undergoing elective cesarean sections at term (37–42 weeks gestation). Women with pre‐existing medical disorders (e.g. diabetes) or pregnancy complications (e.g. gestational diabetes or pre‐eclampsia) were excluded. Omental adipose tissue was collected from six NW (BMI 18.5–24.9 kg/m^2^ recorded prior to 12 weeks gestation) and six OB (BMI ≥ 30 kg/m^2^) women. Myometrial tissue (*N* = 8) was collected from NW women only. An individualized birthweight ratio (IBR) was calculated for each infant using Gestation‐Related Optimal Weight software (Customized Weight Centile Calculator v 5.12/6.2 2009, downloaded from www.gestation.net). Only myometrial biopsies from mothers who delivered appropriate for gestational age infants (IBR 11–89) were included in the study. Additionally, the effect of incubation with leptin (100 nM, 1 h) was assessed in MAs obtained from five women with normal pregnancy outcomes. Maternal demographics, biophysical and obstetric data are presented in Table 1.

**Table 1:** Demographic and obstetric data of study participants

Category	Omentum			Myometrium (*N* = 13)
	Normal weight (*N* = 6)	Obese (*N* = 6)	*P*	
BMI (kg/m^2^)	22.8 (18.9–23.5)	34.6 (30.5–39.7)	** *P* < 0.01	23.6 (17.8–33.0)
Age (years)	33 (23–39)	30 (26–34)	NS	33 (29–40)
Ethnicity				
Caucasian (%)	100	100	NS	84.6
Pakistani (%)	0	0		7.7
Mixed (%)	0	0		7.7
Gravidity	2 (1–4)	2 (1–5)	NS	2 (1–5)
Parity	1 (0–3)	1 (0–4)	NS	1 (0–3)
Smoker (%)	0	0	NS	7.7
Gestational age (weeks)	39^+2^ (38^+6^–41^+2^)	38^+5^ (37^+1^–39^+2^)	NS	39^+0^ (36^+6^–40^+2^)
Birthweight (grams)	3340 (3000–3520)	3649 (3180–4420)	NS	3180 (2840–3920)
IBR	49 (5–66)	67 (36–98)	NS	39 (17–86)

** *P* < 0.01, Mann–Whitney test.

Median (range) is shown unless stated otherwise. BMI, body mass index; IBR, individualized birthweight ratio; NS, not significant.

### General chemicals

Chemicals were purchased from Sigma‐Aldrich unless stated otherwise.

### Omental adipose tissue culture

Omental adipose tissue was collected in tissue collection buffer (154 mM NaCl, 5.4 mM KCl, 1.2 mM MgSO_4_, 1.6 mM CaCl_2_, 10 mM MOPS and 5.5 mM glucose; pH 7.4).^9^ Fragments of tissue were dissected, washed in sterile phosphate buffered solution (10 tablets in 1 L distilled water, equivalent to 137 mM NaCl, 3 mM KCl, 8 mM Na_2_HPO_4_, 1.5 mM KH_2_PO_4_, pH 7.3) and sterile culture medium (containing Medium 199; 10% fetal calf serum; 1% L‐Glutamine–Penicillin–Streptomycin solution [equivalent to 0.2 mM glutamine, 100 IU/mL penicillin, 100 μg/mL streptomycin sulfate]; and 0.25 μg/mL Amphotericin B). Using a similar method to that described previously in placental tissue,^19^ omental adipose tissue explants were cultured on individual Costar Netwell supports (15 mm, 74 μm mesh) for 48 h at 37°C, 20% O_2_, 5% CO_2_. Culturing on Netwells allows the total immersion of adipose tissue in culture medium, preventing it from floating on top of the medium, enables adequate gaseous exchange to maintain tissue viability and avoids damage to tissue when collecting and replacing culture medium. Omental adipose tissue conditioned medium was collected every 24 h (days 1 and 2) and stored at −20°C. After 48 h, omental adipose tissue explants were transferred to 0.3 M NaOH and incubated at 37°C until fully dissolved. Protein content (in mg), used as a proxy for the amount of tissue cultured, was determined by Bio‐Rad protein assay. Day 2 conditioned medium was pooled from cultures of omental adipose tissue collected from six NW pregnant women to produce a NW medium and from six OB pregnant women to produce an OB medium. Medium (control medium) that was not exposed to human tissue was stored at −20°C and used as a control.

### Enzyme‐linked immunosorbent assay

Concentrations of specific hormones, adipokines and cytokines were measured using Quantikine enzyme‐linked immunosorbent assays in the NW and OB media, and in the individual samples that made up the pooled medium. Enzyme‐linked immunosorbent assays for leptin, adiponectin (total including low, middle and high molecular weight), chemerin, IL‐6, IL‐7 (high sensitivity), IL‐8, monocyte chemoattractant protein (MCP1) and TNF‐alpha (high sensitivity) were performed according to the manufacturer's instructions. Hormonal measurements were corrected for explant protein content. There was no difference in protein content between NW (0.43 ± 0.04 mg) and OB (0.66 ± 0.16 mg) samples.

### Wire myography

Myometrial biopsies were taken from the upper lip of the uterine incision and collected in ice‐cold tissue collection buffer (as above).^9^ MAs (377 ± 95 μm diameter, 1–2 mm length) were carefully dissected, mounted on a Danish Myotechnology M610 wire myograph, normalized to an internal diameter of 0.9 of L_13.3kPa_ (luminal pressure ≈ 45 mmHg) and left to equilibrate (37°C, gassed with air/5% CO_2_) in physiological salt solution^9^ (PSS; 119 mM NaCl, 25 mM NaHCO_3_, 4.69 mM KCl, 2.4 mM MgSO_4_, 1.6 mM CaCl_2_, 1.18 mM KH_2_PO_4_, 6.05 mM glucose, 0.034 mM EDTA; pH 7.4), as previously described.^20^ Vessel contractility was assessed using a high potassium solution (KPSS; 11 mM NaCl, 25 mM NaHCO_3_, 120 mM KCl, 2.4 mM MgSO_4_, 1.6 mM CaCl_2_, 1.18 mM KH_2_PO_4_, 6.05 mM glucose, 0.034 mM EDTA; pH 7.4).^9^ MAs were exposed to maximal concentrations of thromboxane A_2_ mimetic, U46619 (10^−5.7^ M) and bradykinin (BK; 10^−5^ M), an endothelium dependent vasodilation agonist to assess endothelium function. Vasodilation to BK was required to reach > 50% of the maximum contraction for the experiment to continue. Following PSS washout, arteries were bathed in 1:8 media (control, NW or OB medium) to PSS solution and left to incubate for 2 h at 37°C. After 2 h, arteries were exposed to incremental concentrations of U46619 (10^−10^–10^−5.7^ M, 6 × 2 min intervals) and incremental concentrations of BK (10^−10^–10^−5^ M, 6 × 2 min intervals). Following PSS washout, KPSS was applied to confirm vessel viability.

Using a similar protocol, the effect of the addition of leptin (100 nM, 1 h) compared to diluent without leptin (control) was assessed in MAs obtained from five women with normal pregnancy outcomes.

### Statistics

Data were analyzed using GraphPad Prism 5.0. Demographic data (median and range) were analyzed by χ^2^ and Mann–Whitney tests. N = number of tissue samples; n = number of vessels. Vessel tone (mN/mm) was converted into active effective pressure (kPa) by normalizing for vessel diameter. Concentration response curves (mean ± standard error of the mean) were analyzed by two‐way repeated measures analysis of variance (a Bonferroni post‐test was used as appropriate). Area under the curve (AUC; arbitrary units), maximum response (contraction or relaxation, V_max_, kPa or percentage) and sensitivity (EC_50_, nM) to each agonist for each artery were calculated. Data for these parameters are presented as median (range) and analyzed using a Friedman test (as appropriate).

## Results

Omental biopsies collected from NW and OB women were closely matched for other maternal characteristics, including ethnicity and smoking status (Table 1).

### Factors in omental adipose tissue conditioned medium

**Figure 1:**
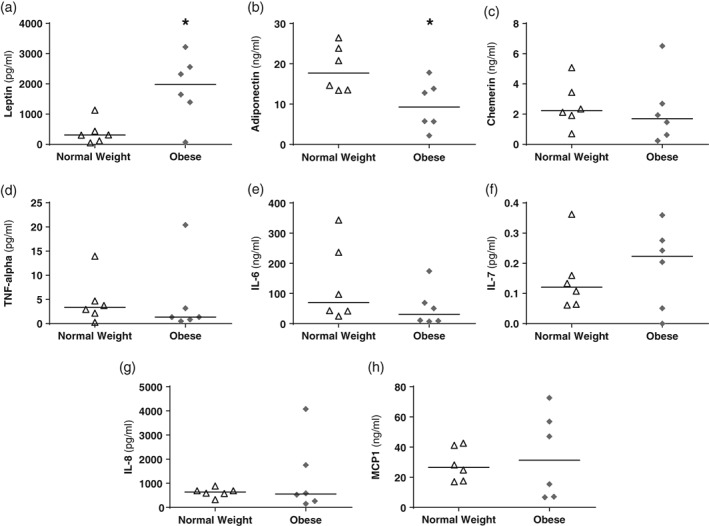
Concentrations of adipokines, cytokines and hormones in the omental adipose tissue medium. (a) Leptin concentrations were higher, while (b) adiponectin concentrations were lower in obese medium compared to normal weight medium. However, there were no differences in the levels of (c) chemerin, (d) tumor necrosis factor (TNF) alpha, (e) interleukin (IL)‐6, (f) IL‐7, (g) IL‐8 and (h) monocyte chemoattractant protein (MCP) 1. *P < 0.05, Mann–Whitney test.

Concentrations of adipokines and cytokines were measured in individual samples of conditioned medium and in the pooled NW and OB media. Leptin concentrations were higher in the individual OB samples compared to the NW samples (*P* < 0.05) (Fig. 1a), and higher in the pooled OB medium (859 pg/mL/mg protein) than in the NW medium (331 pg/mL/mg protein). Adiponectin concentrations were lower in the individual OB samples compared to the individual NW samples (*P* < 0.05) (Fig. 1b) and the pooled NW medium (19 ng/mL/mg protein) had double the concentration of the pooled OB medium (8 ng/mL/mg protein). There were no significant differences in concentrations of chemerin, TNF‐alpha, IL‐6, IL‐7, IL‐8 and MCP1 between individual samples of NW and OB medium (Fig. 1c‐h). The pooled NW medium compared to the OB medium contained: chemerin (3.1 ng/mL/mg protein vs 0.9 ng/mL/mg protein), TNF‐alpha (2.8 pg/mL/mg protein vs 3.7 pg/mL/mg protein), IL‐6 (82 ng/mL/mg protein vs 28 ng/mL/mg protein), IL‐7 (0.19 pg/mL/mg protein vs 0.12 pg/mL/mg protein), IL‐8 (636 pg/mL/mg protein vs 361 pg/mL/mg protein) and MCP1 (36 ng/mL/mg protein vs 22 ng/mL/mg protein). In samples of control medium, only leptin was at a sufficient concentration (8 pg/mL/mg protein) to be detected, none of the other factors reached a measureable level.

### Effect of omental adipose conditioned media on myometrial artery (MA) vasoconstriction

**Figure 2:**
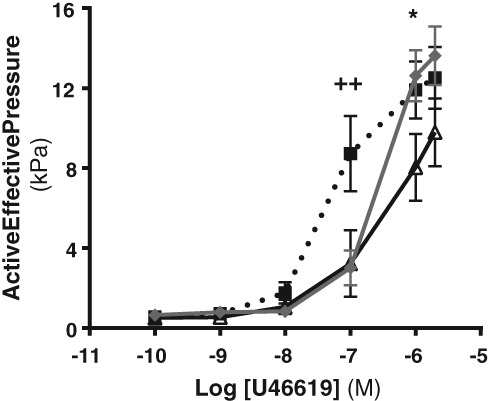
Effect of omental adipose tissue conditioned medium on U46619‐induced vasoconstriction in myometrial arteries. Data are mean ± standard error of the mean. Concentration‐response curves *P* < 0.001, two‐way repeated measures analysis of variance; ++*P* < 0.01 at 10^−7^ M U46619 control medium versus normal weight medium and control medium vs. obese medium; *P < 0.05 at 10^−6^ M U46619 normal weight medium versus obese medium. 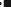, control; 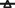, normal weight; 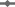, obese.

U46619‐induced vasoconstriction in MAs from NW pregnant women was increased by acute exposure to pooled OB medium compared to pooled NW medium (interaction and effect of media significant; *P* < 0.01) (Fig. 2). In comparison to arteries incubated in control medium, arteries exposed to conditioned media (either NW or OB medium) had significantly lower responses to U46619 (interaction and effect of media significant; *P* < 0.001) (Fig. 2). Differences in the U46619 concentration curves were not accompanied by significant changes in AUC, agonist sensitivity (EC_20_, EC_50_ and EC_80_) or maximum constriction (Table 2).

**Table 2:** Concentration‐curve sensitivity values to the thromboxane mimetic, U46619 and BK in myometrial arteries exposed to control, normal weight and obese media

	Value	Control medium	Normal weight medium	Obese medium	*P*
U46619	AUC	23 (6–32)	11 (5–27)	15 (11–21)	NS
	EC_20_ (nM)	11 (4–184)	126 (2–325)	133 (5–267)	NS
	EC_50_ (nM)	45 (15–738)	505 (6–1301)	530 (20–1068)	NS
	EC_80_ (nM)	181 (59–2950)	2021 (26–5204)	2121 (79–4272)	NS
	Maximum constriction (kPa)	12 (6–18)	10 (2–15)	15 (6–19)	NS
BK	AUC	278 (79–355)	145 (57–283)	233 (30–342)	NS
	EC_20_ (nM)	205 (0–37 160)	47 (1–13 760)	143 (5–25 070)	NS
	EC_50_ (nM)	95 (1–9290)	12 (0–3440)	36 (1–6268)	NS
	EC_80_ (nM)	24 (0–2323)	3 (0–860)	9 (0–1567)	NS
	Maximum relaxation (%)	12 (0–63)	7 (3–34)	8 (0–34)	NS

Data analysis by Friedman test. Data are median (range). AUC, area under the curve; BK, bradykinin; EC, effective concentration; NS, not significant.

### Effect of omental adipose conditioned media on MA endothelial‐dependent vasodilation

**Figure 3:**
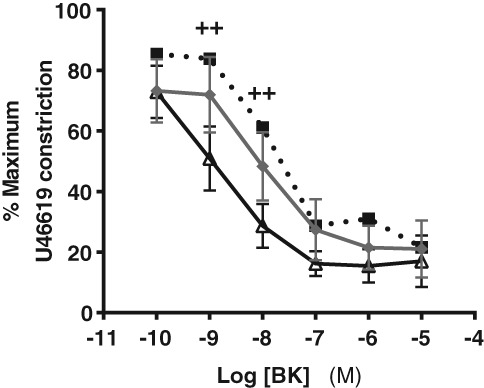
Effect of omental adipose tissue conditioned medium on bradykinin (BK)‐induced vasodilation in myometrial arteries. Data are mean ± standard error of the mean. Concentration‐response curves *P* < 0.001, two‐way repeated measures analysis of variance; ++*P* < 0.01 at 10^−9^ M and 10^−8^ M BK control medium versus normal weight medium. 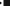, control; 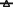, normal weight; 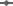, obese.

Endothelial‐dependent relaxation was lower in MAs acutely exposed to pooled OB medium compared to pooled NW medium (effect of media significant, *P* < 0.05) (Fig. 3). However, in comparison to arteries incubated in control medium, BK‐induced vasodilation was greater in arteries exposed to conditioned media (either NW or OB medium) (*P* < 0.0001) (Fig. 3). There were no significant differences in AUC, maximum relaxation or agonist sensitivity between myometrial arteries in control, NW or OB medium (Table 2).

### Effect of leptin incubation on MA constriction and endothelial‐dependent vasodilation

**Figure 4:**
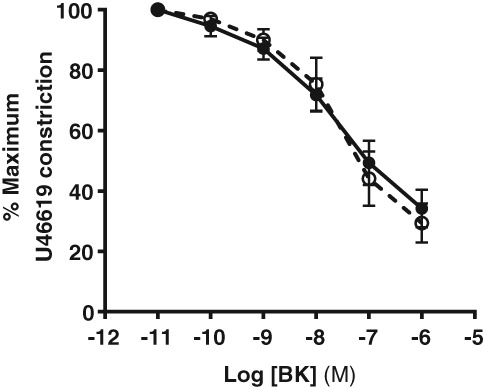
Effect of leptin pre‐incubation on bradykinin (BK)‐induced vasodilation in myometrial arteries. Data are mean ± standard error of the mean. Concentration‐response curves *P* > 0.05, two‐way repeated measures analysis of variance. 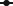, control; 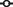, leptin 100nm.

MA constriction was not affected by acute incubation with 100 nM leptin (for 1 h, data not shown). Endothelial‐dependent relaxation was not significantly affected in MAs by incubation with leptin (Fig. 4).

## Discussion

Obese medium (conditioned medium collected after culturing omental adipose tissue taken from OB pregnant women) contained altered concentrations of adipokines, namely leptin and adiponectin, compared to NW medium (conditioned medium from cultures of omental adipose tissue from NW pregnant women). Levels of other factors, such as cytokines, previously identified as abnormal in obesity, were similar in NW and OB media. MA function was altered by acute exposure to omental adipose tissue conditioned medium irrespective of BMI; contraction to U46619 was reduced and relaxation to BK improved compared to MA function in the presence of the control medium. Between the two conditioned medium conditions, OB medium versus NW medium exposure increased U46619‐induced vasoconstriction and reduced vasodilation to BK in MAs collected from NW women. Using arteries from NW women excluded the potential complicating effects of maternal obesity on vascular reactivity and offered us a model to try and mimic the responses previously observed in MAs from OB women.^9^

Exposure to omental adipose tissue significantly altered the composition of the control medium. Of all the factors measured in the current study only leptin, at a very low concentration, was present in the control medium. This demonstrates that omental fat releases adipokines and cytokines into the surrounding environment, which, if released into the circulation *in vivo*, have the potential to alter vascular function. As expected from previous studies of obesity, concentrations of leptin were higher, and adiponectin lower, in OB compared to NW medium.^15^, ^21^, ^22^, ^23^, ^24^ Exposure to NW and OB media was associated with changes in MA function in comparison to the control medium, indicating that the presence of adipokines in the culture medium modulates vascular reactivity. The difference in leptin and adiponectin concentrations in the NW and OB media may account for the different responses observed in MAs.

Leptin has been shown to induce vasodilation in a variety of vessel types. In rats, leptin stimulates vasodilation through endothelial‐dependent mechanisms,^25^, ^26^ via NO or endothelial‐derived hyperpolarizing factor, and by endothelial‐independent mechanisms via membrane hyperpolarization following the activation of ATP‐sensitive potassium channels.^27^, ^28^ However, chronic leptin exposure induces sustained increases in blood pressure and heart rate in rodents;^29^ increased sympathetic nervous system activation has been suggested as a possible mechanism by which this may be achieved.^30^ Here, we showed that acute leptin exposure did not directly alter endothelial dependent function in MAs isolated from women with uncomplicated pregnancies. The effect we have noted with acute exposure to OB medium is therefore unlikely to be a result of increased leptin levels, however it remains a possibility that high levels of circulating leptin (as noted in chronic obesity) may induce dysregulation of MA reactivity.

The presence of adiponectin in conditioned medium may have contributed to the observed changes in vascular reactivity. Adiponectin is a vasoprotective factor that induces vasodilation by activating endothelial cell NO production;^31^, ^32^ higher concentrations in the NW versus OB medium may further alter arterial contraction while aiding relaxation to BK. Interestingly, adiponectin has also been demonstrated to directly affect vascular smooth muscle cells (induces relaxation);^33^ however, an assessment of adiponectin receptor expression and activation (e.g. with AdipoRon) to determine whether a similar mechanism(s) is present in MAs was beyond the scope of this initial study. Interestingly, *in vitro*, adiponectin has been found to inhibit the activity of TNF‐alpha in endothelial cells,^34^ and TNF‐alpha has been shown to increase leptin release from adipocytes through post‐translational modifications.^35^ Although a direct link has not yet been made, lower adiponectin concentrations in obesity may indirectly contribute to increased maternal circulating leptin levels.

Maximal contraction to U46619 in the presence of control or conditioned media was similar in magnitude to our previous observations in MAs from NW and OB pregnant women.^9^ However, acute exposure to OB medium had the opposite effect to the hypothesis and induced an upward shift in the U46619‐concentration response curve, while NW medium appeared to impair U46619‐induced contraction. These responses may be the result of aberrant levels of adipokines, as discussed above. Direct comparisons cannot be made between our published BK‐induced vasodilation data and that of the current study, as MAs were previously preconstricted to 80% of the maximal U46619‐induced contraction, while in the current study, relaxation to BK was performed in the presence of a maximal concentration of U46619. Because of the relatively small amounts of medium available for collection and pooling, this was unavoidable, as it was not possible to change the medium between concentration response curves. Ostensibly, exposure to OB compared to NW medium reduced BK‐induced vasodilation in MAs from NW pregnant women and although the upward shift in the BK concentration response curve did not reach the same extent as our previous data, it did mimic the functional difference between arteries from NW and OB pregnant women.

Changes in adipokine concentrations, resulting from an increase in central fat stores, represent one possible mechanism promoting impaired vascular function in obesity. Other studies, however, suggest that altered maternal plasma lipid composition and oxidative stress may link impaired vascular function and obesity. When central fat stores increase in obesity there are changes in plasma lipid composition as fatty acids are released into the circulation.^36^ This causes lipotoxicity, the accumulation of fatty acids in non‐adipose tissues, which results in intracellular oxidative stress and the production of oxidized lipids.^37^, ^38^ Published data indicate that increased levels of circulating fatty acids and oxidized lipids are associated with vascular endothelial dysfunction.^39^ Adaptations in normal pregnancy include lipid mobilization and insulin resistance. In OB pregnancies, these events are increased and, as insulin can mediate endothelial function by altering lipolysis and circulating fatty acid concentrations, this may explain why OB women have impaired vascular endothelial function.^38^, ^39^ The current study focused on the potential role of adipokines in vascular dysfunction, thus the lipid composition of the conditioned medium was not examined. Further work is required to better characterize conditioned medium with respect to the identity and concentration of lipids and other adipokines; a metabolomic screen of NW and OB media will be most informative in this regard. Our subsequent experiments will focus on determining which specific adipokines or lipids (alone or in combination) lead to altered MA function in OB pregnant women.

Comparing omental adipose conditioned medium to maternal circulating levels measured previously in pregnancy,^13^, ^21^, ^40^, ^41^ the concentrations of leptin, adiponectin and chemerin were 100–1000 fold lower, concentrations of IL‐6, IL‐8 and MCP1 were 1000 fold higher and concentrations of TNF‐alpha and IL‐7 were similar. Variability in these concentrations may arise from the inevitable tissue damage during dissection; however, conditioned medium from day 2 (rather than from day 1) was pooled to prevent factors released from damaged cells in the first few hours of culture having a confounding influence on the results. Adipose tissue floats in medium, which can make it difficult to culture and can introduce variability in results. To prevent this, a previous study used a new ‘bottom culture’, which involved embedding non‐pregnant omental adipose tissue in medium containing hydrogel, starving the tissue for 12 h prior to incubating it with serum collected from pregnant women and examining the effects on inflammatory and adipogenic activities.^42^ Significant changes were observed in the expression of genes regulating immune responses, oxidative stress and lipid metabolism in the non‐pregnant adipose tissue when exposed to serum from pre‐eclamptic compared to healthy pregnant women. To avoid possible confounding effects of embedding or starving the tissue, Netwells were used in the current study to maintain conditions relatively consistent between cultures. Culturing on Netwells enables adipose tissue explants to sit (without floating) on the Netwell mesh, immersed in culture medium with adequate gaseous exchange to maintain tissue viability, and with little disturbance during the culture period (i.e. during culture medium change). Differences observed in adipokine concentrations are therefore most likely caused by the small amount of tissue used in the explant culture: either there was not enough tissue to produce the same concentration or the small size of the explant may have caused an increased amount to be released (as observed in placental explants).^43^ The results of the current study demonstrate that omental adipose tissue explants produce detectable concentrations of adipokines that appear to be sufficient (in an *in vitro* model with all its caveats) to induce aberrant vascular function in vessels that would normally be distant from the site of production, that is, central fat. Adipose tissue was only cultured for a relatively short period (48 h) to avoid any changes in adipocyte and stromal‐vascular cell morphology, as shown previously,^44^ which may promote the production of different endocrine factors.

Although these data suggest that a central fat store can produce adipokines that influence the behavior of a ‘remote’ blood vessel, it is important to note that there are other fat stores that may release factors that have more localized effects on vascular function in OB mothers. Perivascular adipose tissue (PVAT), a local deposit of adipocytes around blood vessels (which was not investigated in the current study), releases factors locally that can promote an anti‐contractile effect on vascular function.^45^ However, in obesity this does not happen as aberrant concentrations of factors, such as TNF‐alpha, downregulate endothelial NO synthase activity, causing endothelial dysfunction.^46^ Following bariatric surgery, levels of adipokines, such as adiponectin and NO, are normalized and PVAT anti‐contractile effects restored. Subsequent blocking of adiponectin in small arteries dissected from gluteal subcutaneous fat samples inhibit the vasodilatory effects of PVAT, indicating that adiponectin along with NO has a role in the control of arterial function.^47^ Studies of obesity demonstrate that if central stores reach their capacity to store fat, there is overspill of fatty acids into the circulation, which then accumulates in other organs, for example, the liver, muscle and heart, and cause cellular dysfunction (lipotoxicity).^38^ One previous study determined that there are normally some fat deposits in uterine stroma,^48^ but whether these increase as a result of lipotoxicity in OB women is unknown. The balance of adipokine effects from central or local (PVAT or uterine) stores is likely to be important in MA function and future studies should examine the relative contributions of both. In addition, magnetic resonance imaging scans of the uterus could be used to determine whether fat accumulates in OB women during pregnancy.

## Conclusion

The current study demonstrated that omental adipose tissue taken from OB mothers released aberrant concentrations of leptin and adiponectin compared to that from NW women. Adipokines in conditioned medium are associated with changes in MA function, that is, in vessels from vascular bed spatially distinct *in vivo.* Aberrant vasoactive adipokine production or secretion from central fat stores may therefore promote the systemic, utero‐placental and feto‐placental vascular dysfunction evident in OB mothers. The resultant behavior of uterine blood vessels in OB women, which are key for the adequate perfusion of the uteroplacental vasculature, is likely to be a balance between the effects of centrally derived, circulating adipokines and local factors (e.g. shear stress, oxygenation, PVAT). As the prevalence of obesity is increasing in the general and pregnant populations, it is important to understand the underlying mechanisms of impaired vascular function, which may contribute to the susceptibility of OB mothers to poor pregnancy outcomes.

## Disclosure

No author has any potential conflict of interest.
